# Editorial: Non-coding RNA assisted plant response to stresses

**DOI:** 10.3389/fpls.2023.1276427

**Published:** 2023-09-19

**Authors:** Ning Jiang, Jun Cui

**Affiliations:** ^1^ Hunan Agriculture Product Processing Institute, Hunan Academy of Agricultural Sciences, Changsha, Hunan, China; ^2^ Hunan China State Key Laboratory of Developmental Biology of Freshwater Fish, Hunan Provincial Key Laboratory for Microbial Molecular Biology, College of Life Science, Hunan Normal University, Changsha, Hunan, China

**Keywords:** non-coding RNAs, abiotic stress, biotic stress, function, plant

With the increases in the temperature of Earth’s surface, drought is occurring more frequently, and excessive fertilizer application has led to soil salinization during crop production. Additionally, plants are often harmed by pathogens during their growth and development. These abiotic and biotic stresses threaten crop production and food supplies. In addition, increasing the survival rate of plants in saline-alkali soils and deserts is also beneficial for increasing the land utilization rate. Therefore, determining how plants adapt to severe biotic and abiotic stresses in order to improve their resistance and tolerance is urgently necessary.

Non-coding RNAs (ncRNAs) are a class of functional RNAs transcribed from the genome with the assistance of RNA polymerases. The powerful regulatory roles of miRNAs are well known worldwide. The ncRNAs include small RNA (sRNA) and long non-coding RNA (lncRNA). Recent research has showed that plant ncRNAs act in the regulation of abiotic and biotic stress responses. This Research Topic encompasses three original research articles and one mini review article.

sRNA includes siRNA and miRNA, which have been reported to transfer between species and have biological function. Wu et al. identify 21 *Botrytis cinerea*-induced sRNAs from tomao as the regulator candidates through bioinformatics and qRT-PCR method. Among them, miR396a-5p, siR3 and siR14 can target virulence genes of *B. cinerea*. Meanwhile, through gene silences analysis, it shows that these 3 sRNAs could target the virulence factor genes of *B. cinerea*, and spore gemination is also inhibited. These results suggest that these *B. cinerea*-induced tomato sRNAs could inhibit *B. cinerea* infection through targeting the virulence factors. In addition, double strand (ds)-miR396a-5p is more effective to inhibit virulence of *B. cinerea* than single strand (ss)-miR396a-5p, showing the decreased necrosis area in ds-miR396a-5p-treated leaves after *B. cinerea* infection. All of these suggest sRNA plays an important role in plant-pathogen interaction and provide a new direction for the use of plant sRNAs to control pathogens.

MiR482/2118, a miRNA superfamily is involved in plant resistance to pathogens by targeting resistance gene *NBS-LRR*. Besides, other molecular mechanisms associated with miR482/2118 is also found to affect plant resistance. Liao et al. review these new insights. Firstly, miR482/2118-3p and -5p have different expression patterns in pathogen infection and target different genes, but they coregulate plant resistance. Secondly, miR482/2118 trigger the phasiRNA production by cleave *NBS-LRR* genes and lncRNAs, which regulate plant resistance. Thirdly, some lncRNAs with binding sites of miR482/2118 can act as endogenous target mimics decoying miR482/2118, enhancing target gene *NBS-LRR* levels. Last, lncRNAs locates on the antisense sequence of *MIR482/2118* gene and suppresses the expression of *MIR482/2118* gene to regulate mature miR482/2118 levels. All these new insights provide a comprehensive view describing the molecular mechanism associated with miR482/2118 in the plant immune system.

In addition to biotic stress, ncRNAs are also involved in the process by which plants tolerate abiotic stress. Swida-Barteczka et al. find that hvu-miRNA172b-3p cleaving the four target genes APETALA2(AP2)-like genes, acts in barley response to drought stress. While hvu-miR172b-5p originating from a single precursor with hvu-miRNA172b-3p targets trehalose-6-phosphate synthase gene, which is a key enzyme in flowering pathways. Therefore, the hvu-miRNA172b-5p and hvu-miRNA172b-3p lead to osmoprotection and accelerated flowering through different response patterns and regulation different target genes during drought.

In addition to miRNAs, an important class of lncRNAs, natural antisense transcripts (NATs) affect gene expression at the transcriptional or post-transcriptional level. Jin et al. identify 36,317 NAT pairs from *Arabidopsis thaliana* through strand-specific RNA sequencing. Among them, 5,536 can response to heat stress. After heat stress, the positive expression correlation of heat-regulated NAT pairs (coding genes and NATs) are more than the negative correlation NAT pairs. The heat-regulated NAT pairs are enriched with histone modification. Histone levels of NAT genes are associated with H3K27me3 and H3K4me3 makers. Furthermore, it is found that many NAT genes is the precursors of nat-siRNAs, maybe producing siRNAs, and these sRNAs exist in the promoter regions for NAT genes.

In brief, this Research Topic highlights the latest findings on the role of non-coding RNAs in plant resistance and tolerance to biotic and abiotic stresses. The molecular mechanisms of non-coding RNAs in biotic and abiotic stresses are reviewed and the regulatory or interaction relationships between different non-coding RNAs are elucidated. The theoretical achievements in this Research Topic can improve and perfect the molecular mechanism of non-coding RNA in response to stress and lay a foundation for the application of non-coding RNA in plant resistance breeding.

## Author contributions

NJ: Data curation, Writing – original draft. JC: Funding acquisition, Supervision, Validation, Writing – review & editing.

